# Long-Term Outcomes of the Good School Toolkit Primary School Violence Prevention Intervention Among Adolescents: Protocol for a Nonrandomized Quasi-Experimental Study

**DOI:** 10.2196/20940

**Published:** 2020-12-07

**Authors:** Louise Knight, Lydia Atuhaire, Elizabeth Allen, Sophie Namy, Katharina Anton-Erxleben, Janet Nakuti, Angel Faridah Mirembe, Mastula Nakiboneka, Janet Seeley, Helen A Weiss, Jenny Parkes, Chris Bonell, Dipak Naker, Karen Devries

**Affiliations:** 1 London School of Hygiene and Tropical Medicine London United Kingdom; 2 Medical Research Council/Uganda Virus Research Institute and London School of Hygiene and Tropical Medicine Uganda Research Unit Entebbe Uganda; 3 Raising Voices Kampala Uganda; 4 Medical Research Council Tropical Epidemiology Group London School of Hygiene and Tropical Medicine London United Kingdom; 5 University College London Institute of Education London United Kingdom

**Keywords:** violence, long-term follow-up, whole-school, intervention, adolescents, Uganda, resilience, non-randomised

## Abstract

**Background:**

Violence against children in schools is a global public health problem. There is growing evidence that school-based interventions can be effective in reducing violence against children in schools. However, there is little evidence on the long-term impact of such interventions. The Good School Toolkit, developed by Raising Voices, a Uganda-based nonprofit organization, is a whole-school violence prevention intervention that aims to change the operational culture of primary schools. In 2014, the Good School Toolkit was evaluated through a cluster randomized controlled trial (Good Schools Study) and found to reduce teacher-to-student and student-to-student violence.

**Objective:**

This protocol describes quantitative analyses to explore long-term outcomes of the Good School Toolkit intervention among adolescents in Uganda, including the extent to which it is associated with peer-violence victimization (primary outcome) and peer-violence perpetration, intimate-partner violence, acceptance of teacher-violence, equitable gender attitudes, agency, self-regulation, peer connectedness, social assets, psychological assets, and retention in school (secondary outcomes).

**Methods:**

This is a nonrandomized quasi-experimental 4-year follow-up study of adolescents who attended the 42 Good Schools Study primary schools in 2014; 21 schools initiated the Good School Toolkit intervention during the trial from 2012, and 19 schools initiated the intervention after the trial (during the later delivery phase) from 2015; 2 schools did not implement the intervention. Students in the final school grade (Primary 7) during 2014 of the 19 primary schools in the later delivery phase are expected to have left school prior to toolkit delivery in 2015. Wave 1 data were collected in 2014 from 3431 grade Primary 5 to Primary 7 school students aged 11-14 years; these students were followed up in 2018-2019 when aged 16-19 years and invited to participate in the Wave 2 survey. Data were collected in face-to-face interviews by trained Ugandan field researchers. Toolkit exposure groups are defined as exposed during the Good Schools Study trial (from 2012), as exposed during later delivery (from 2015), or not exposed including those expected to have completed Primary 7 prior to later delivery or from the 2 schools that did not implement the toolkit. Associations between outcomes at Wave 2 and toolkit exposure groups will be analyzed using mixed-effect multivariable logistic and linear regression models for binary and continuous outcomes, respectively. This analysis is exploratory and aims to generate hypotheses on if, and under what circumstances, the toolkit influences later adolescent outcomes.

**Results:**

Data collection was completed in August 2019.

**Conclusions:**

To our knowledge, this is the first long-term follow-up study of adolescents exposed to a school-based violence-prevention intervention in sub-Saharan Africa. If the intervention reduces violence and improves other outcomes in later adolescence, then this study supports primary school interventions as key to achieving long-term population impacts. The pattern of effects will inform where reinforced or additional interventions are needed.

**International Registered Report Identifier (IRRID):**

DERR1-10.2196/20940

## Introduction

### Background

Globally, over 1 billion children experience violence every year [1]. In Uganda, 75% of 18- to 24-year-old individuals report violence in their childhood [2], and over half of ever-married women report physical violence from an intimate partner in their lifetime [3]. Violence against children that occurs in school is a global public health problem that takes many forms. It may include physical, emotional, and sexual violence and involve different perpetrators and power dynamics [4]. In Uganda, similar to in other resource-constrained settings, the majority of primary school students have experienced physical violence from a teacher, and almost half have experienced physical, emotional, or sexual violence from a fellow student [5-7]. Negative health, social, and economic outcomes among those who experience violence in childhood have been well described [8]. Furthermore, experiencing violence as a child may lead to a “cycle of violence” with increased aggressive behavior and greater risk of boys perpetrating violence and girls experiencing intimate-partner violence later in life [9]. Uganda’s Demographic and Health Survey 2016 indicated that, nationally, 84% of primary school–aged children attend some primary school [3], and 60% of those who complete primary school transition to secondary education [10]. The extent and consequences of violence call for urgent effective violence prevention strategies that can protect against childhood violence and reduce the risk of future violence by supporting positive transitions through adolescence into adulthood [11,12].

### Long-Term Effects of School-Based Violence Prevention Interventions

Evidence from high-resource settings suggest that some school-based violence-prevention interventions show promising long-term effects on violence outcomes 3 or more years later [13,14]: Safe Dates, delivered to the eighth-grade students in the United States, reduced dating violence 4 years later [15]; Learning Together, in UK secondary schools, reduced bullying victimization at 36 months [16]; Gatehouse Project, a social-inclusion intervention in Australian secondary schools, reduced risky behavior (a composite measure that included interpersonal violence) after 4 years [17]; Aban Aya Youth Project, a social-emotional program delivered to Grade 5 to 8 students in the United States, reported a reduced rate of increase in violent behavior among boys over 4 years [18]; and Positive Action, a multiple-risk behavior intervention in Hawaii and US eliminatory schools, which reported reduced violent behavior after 3 to 6 years [19,20]. The majority of these interventions have been delivered in secondary schools through a variety of approaches, and outcomes are assessed when students are still in school [13]. Effective pathways from primary school interventions to long-term violence reductions are less understood in resource-constrained contexts. To our knowledge, no whole-school intervention that acts on reducing teacher-to-student violence has been evaluated in terms of long-term impacts after adolescents transition out of primary school.

### The Good School Toolkit Intervention

The Good School Toolkit, a violence against children–prevention intervention, was developed by Ugandan nonprofit organization Raising Voices and is freely available for download on their website [21]. It is a complex school-wide intervention that addresses multiple actors and behaviors and was designed to be locally adapted. The intervention is school-led through 2 appointed teacher and student protagonists. Materials are provided along with over 60 interactive and accessible activities that engage the whole school as they sequentially complete the 6 core steps (Textbox 1).

**Good School Toolkit steps [21].**

Step One: Your Team & Network

- Schools identify key protagonists at school and create their Good School Committee to build school-wide support for the process (precontemplation)

Step Two: Preparing for Change

- Baseline measurements gather information on each schools’ starting point, and school leaders cultivate interest among parents, the community and local education officials (contemplation)

Step Three: Good Teachers & Teaching

- A school-wide reflection on teacher-student relationships provides a renewed sense of teacher roles, increased professional support, and new approaches for positive student engagement (preparing for action)

Step Four: Positive Discipline

- Schools reflect on how violence manifests and establish a new school culture by exploring positive disciplinary methods to create students who believe in themselves (action).

Step Five: Good Learning Environment

- Schools reflect on what a good learning environment looks like and work with all stakeholders to foster a psychological sense of safety and inclusion (maintenance of action)

Step Six: Good Administration & the Future

- The work of the preceding steps is celebrated and consolidated through reflection and transfer of leadership to the school administration (consolidated gains)

**Figure 1:**
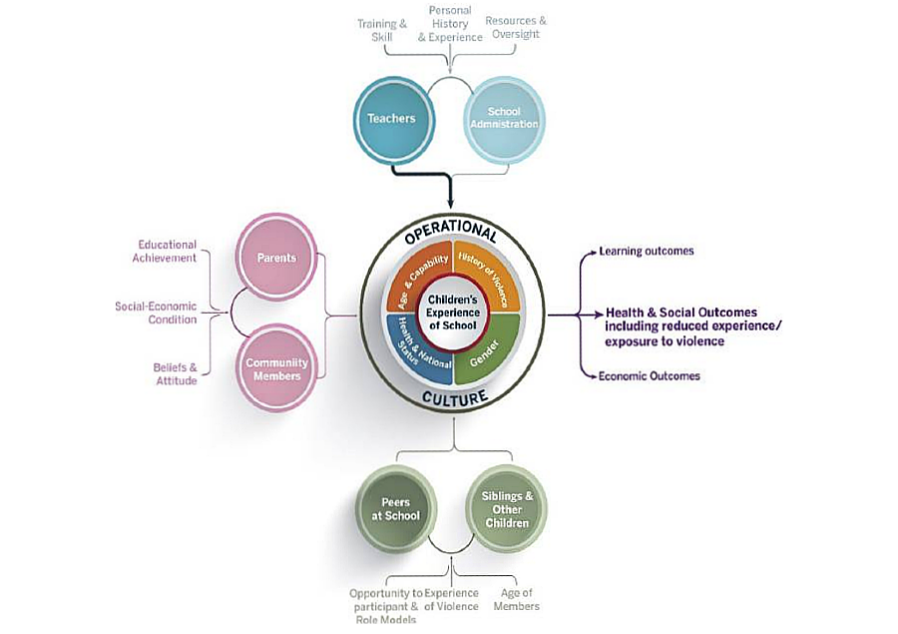
Good School Toolkit programmatic theory of change.

Figure 1 summarizes the toolkit programmatic theory of change [22]. The intervention aims to positively transform operational culture within primary schools. Raising Voices conceptualizes school operational culture as consisting of 3 domains drawn from Moos’ [23] interpretation of the social-ecological framework: (1) psychological, referring to attachment, belonging, identification with—and attitudes toward—the school; (2) relational, referring to interpersonal relationships between teachers and students and between students; and (3) structural, referring to policies, administrative infrastructure, and capacity [24].

The Good School Toolkit is one of the few school interventions with a demonstrated impact of reducing school violence in a resource-constrained setting, including teacher-to-student violence [25]. The Good Schools Study included a cluster randomized controlled trial [26,27], qualitative study [28], process evaluation [29], and economic evaluation [30]. During the trial, conducted in Luwero district in Uganda from 2012-2014, the toolkit was delivered to 21 intervention schools that were compared with 21 wait-list control schools. The trial found that the toolkit reduced the relative risk of past-week teacher-to-student physical violence by 42% [27]. Secondary trial analysis found that the toolkit reduced teacher-to-student past-week physical, emotional, or sexual violence (adjusted odds ratio [OR] 0.41, 95% CI 0.26-0.64) and any student-to-student past-week physical, emotional, and sexual violence (adjusted OR 0.70, 95% CI 0.51-0.96) [31,32]. Subsequently (from 2015), the toolkit was delivered to 19 of the wait-list control schools. While the intervention remained the same (see Table 1), during later delivery the intervention was implemented by *resource persons*, trained and supported by Raising Voices, rather than the Raising Voices program team.

**Table 1:** The Good School Toolkit intervention delivered during the trial and later delivery to study schools.

TIDieR [33] guideline	Description of the toolkit
Brief name	Good School Toolkit
Why	The goal of the toolkit was to transform the operational culture at the school level such that violence against children is prevented. The toolkit draws on the Transtheoretical Model [34] and contains behavior change techniques that have been shown to be effective in a variety of fields [35] and have been included in interventions to change teacher behavior in primary schools [36,37] and reduce perpetration of intimate partner violence [38].
What-materials and content	The toolkit materials consist of books, booklets, posters, and facilitation guides for about 60 different activities. These activities are related to creating a better learning environment, respecting each other, understanding power relationships, using nonviolent discipline, and improving teaching techniques. All materials are publicly available online. Specific behavior change techniques for staff, students and administration included setting school-wide goals, developing action plans with specific dates for deliverables, encouraging empathy by facilitating reflection on experiences of violence, providing new knowledge on alternative nonviolent discipline, and providing opportunities to practice new behavioral skills. Schools were encouraged to self-monitor their progress according to their action plans. Reinforcement of new information and ideas, feedback on progress, and modeling of new techniques and behaviors were provided by visits from the Raising Voices team and also within the school by protagonists to their peers as they gained new knowledge and skills. Children actively participated and formed committees and groups related to different activities. Schools rewarded the successful achievement of their goals and action plan deliverables by creating celebrations. Social support for behavior change was also created because the intervention engaged multiple groups within a school (teachers, administration, students, and also parents) to change ideas and attitudes.
What-procedures	Following a school’s agreement to participate, an inception visit of 2 hours was held, where Raising Voices introduced the toolkit to all school staff. Once the schools were committed to implementing the toolkit, at least 2 staff protagonists were identified who attended a 3-day residential workshop. During this workshop, the protagonists became familiar with the toolkit and developed an action plan for their school. Raising Voices staff members then provided direct one-on-one support and mentorship to key staff protagonists students and at least 2 key student protagonists in each school to carry out the action plan. The toolkit itself has 6 steps, which were designed to be implemented in sequence to guide schools through a systematic process of change. Protagonists could choose which activities they implemented but should complete a minimum number from each step before moving on to the next.
Who provided	Delivery to trial schools: Raising Voices program staff members were trained facilitators and advocates and had received approximately 100 hours of training with individualized coaching support to understand the ideas and content of the toolkit. Later delivery to schools: Raising Voices resource persons were experienced consultants, usually working within the education sector who received at least 20 hours of training and then subsequent individualized coaching support by Raising Voices program staff to understand the ideas and content of the toolkit and how to provide support to school-based protagonists. The key protagonists in each school were 2 teachers who receive 3-day residential workshop based training and ongoing support.
How	During the intervention, Raising Voices staff or resource persons provided direct one-on-one support in the form of in-person visits and telephone calls to staff protagonists, and in-person visits to student protagonists. Staff and student protagonists conducted face-to-face activities with other staff and students in their school, mainly in groups. Children and staff members encouraged others to form, lead, and join groups for various intervention activities.
Where	Activities with students and staff were conducted in schools. Some activities involved creating a better school environment by painting murals on school walls and hanging codes of conduct in visible places; however, the intervention does not require any physical infrastructure.
When and how much	Raising Voices staff made in-person visits to protagonists in each school quarterly and telephoned school staff members approximately monthly, although this varied slightly depending on need. The toolkit itself was designed to be implemented flexibly, and there was no prescribed number of activities or set schedule upon which they should be implemented. However, schools should proceed in sequence and conduct a minimum number of activities, which depends on the stage, prior to progressing to the next stage.
Tailoring, modifications, fidelity	The toolkit was specifically designed to be flexible and adaptable to individual schools. To ensure more uniformity during the trial, Raising Voices staff visited each school at least once per school term, conducted a 3-day residential workshop with all teacher protagonists, met with protagonists to review progress after 1 to 2 terms of implementation, and held meetings with protagonists so they could learn from each other.

### Long-Term Effects: Conceptual Framework

**Figure 2:**
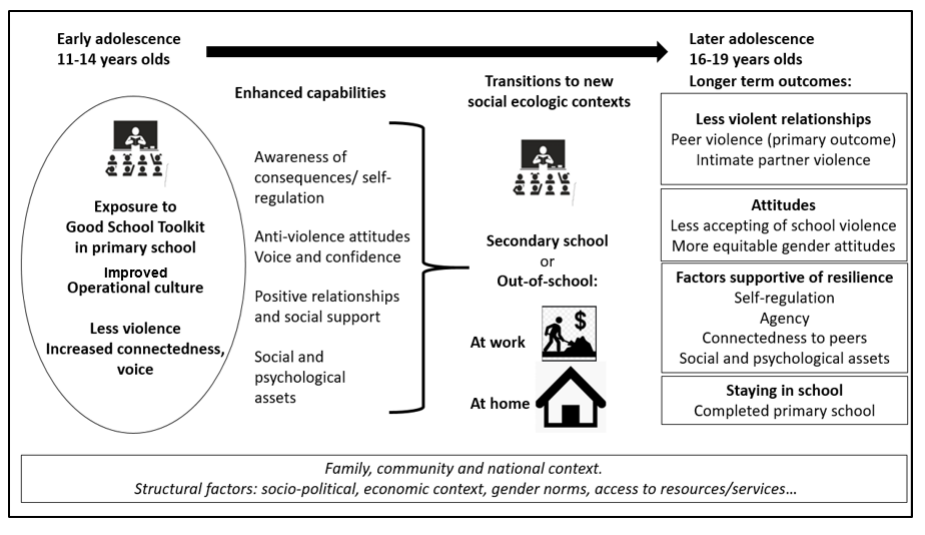
Schematic illustrating the toolkit intervention long-term effects conceptual framework.

A schematic that illustrates mechanisms through which the Good School Toolkit may achieve long-term effects is shown in Figure 2. The Good Schools Study’s quantitative and qualitative findings suggested that the toolkit intervention improved the schools’ operational culture and enhanced student capabilities, including improvements in student-teacher relationships; fostered attitudes that were less accepting of teacher violence; enhanced emotional support from peers; allowed enhanced school-connectedness; and improved self-regulation, voice, and motivation [28,31,39-41]. We hypothesize that students who experienced the toolkit intervention during their primary education (ie, were exposed to a supportive culture and less violence in a safer school environment) are less likely to drop out of school and therefore are retained in the education system for longer. Furthermore, we hypothesize that students’ enhanced capabilities—brought about by the toolkit intervention—can be retained through the transition out of primary school into new environments, equipping adolescents with individual skills supportive of resilience. Through these positive changes brought about by exposure to the toolkit intervention, we hypothesize that adolescents will have attitudes that are less accepting of violence, will be more gender-equitable, and will be less likely to perpetrate or experience peer and intimate partner violence in later adolescent relationships.

Adolescents’ interactions within supportive social ecologies are important for both positive development and resilience [39,42]. Environments outside of primary school are also likely to influence adolescents’ trajectories, in particular, family support, economic pressures, and violence experienced outside of school. As adolescents transition out of primary school, experiences in new secondary schools, the workplace, and domestic contexts are expected to affect adolescents’ outcomes. While enhanced capabilities may support Ugandan adolescents in navigating risk of violence and collectively advocate for change, this is largely limited by gender-related vulnerabilities within shifting risk environments [43]. In addition, adolescents’ negotiations and need for support, resources, and services may not be met by family, school, workplace, or the community systems, which are all subject to wider structural factors [44]. Consequently, the economic and sociopolitical contexts, gender norms, and access to safe secondary education or employment are expected to influence adolescents’ opportunities for positive adaptations and agency in Uganda [45].

### Research Questions

We aim to evaluate long-term effects of the Good School Toolkit, specifically whether exposure to the toolkit intervention in early adolescence is associated with (1) less violent relationships in later adolescence, including peer-violence victimization (primary outcome), peer-violence perpetration, and intimate-partner violence victimization among female adolescents and perpetration among male adolescents; (2) lower acceptance of teacher violence and more equitable gender attitudes in later adolescence; (3) factors supportive of resilience in later adolescence (enhanced agency, self-regulation, peer connectedness, and social and psychological assets); and (4) increased retention in school.

## Methods

### Design

The proposed quantitative analysis makes use of the variation in the Good School Toolkit intervention delivery to create a nonrandomized quasi-experimental design, comparing outcomes across levels of toolkit exposure. This analysis is exploratory and aims to generate hypotheses on if, and in what circumstances, the toolkit influences later adolescent outcomes. To better understand how context and structural factors may influence later outcomes, further quantitative and qualitative analysis is planned and will be informed by this analysis.

### Sampling, Intervention Delivery, and Survey Assessments

**Figure 3:**
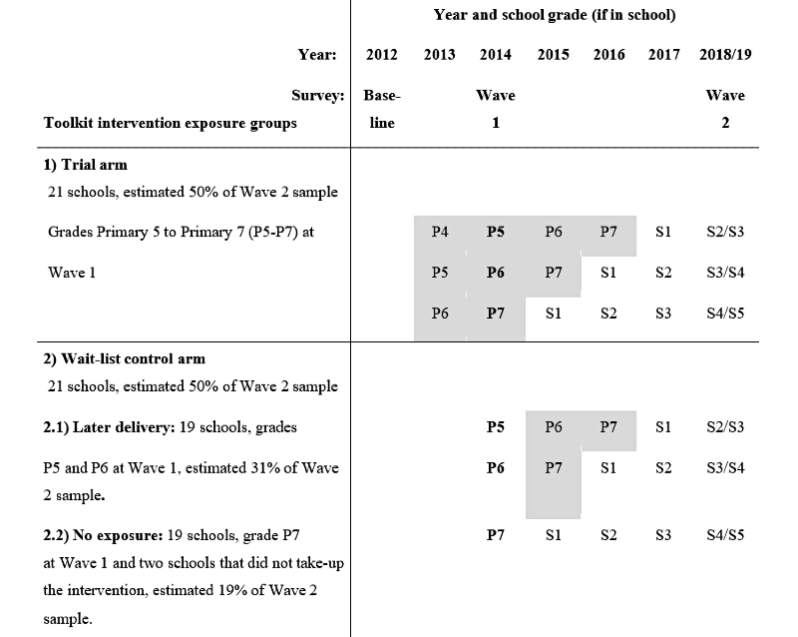
Good School Toolkit exposure by calendar year and across school grade. Boldface school grade denotes school grade at Wave 1 survey. Grey shade indicates students’ expected Toolkit intervention exposure, showing grades and duration of exposure. *At Wave 1 all cohort adolescents were students in primary school grades P5-P7 (approx. aged 11-14) and at Wave 2 cohort adolescents are expected to be either in secondary school grades (S2-S5) or no longer attending a school (approx. aged 16-19).

The original Good Schools Study trial sampling is fully described elsewhere [26]. In summary, we used the 2010 list of 268 government and nongovernment primary schools in Luwero district, obtained from the Ministry of Education. Eligibility criteria were school size (>40 Primary 5 students) and having no existing interventions. Of 151 eligible primary schools that were identified and stratified into 3 groups based on the student male to female ratio (>60% girls, >60% boys, or approximately even), 42 schools were randomly selected proportional to the size of stratum. All selected school headteachers agreed for their school to participate. After the trial baseline survey in 2012, stratified block randomization was used to allocate schools to receive the intervention immediately or to be wait-listed to receive the intervention after the end of the trial (Figure 3). Stratified randomization was used to ensure balance regarding the following key factors: baseline violence, whether the school was urban or rural, and a qualitative assessment of the likelihood of attrition throughout the trial. For both the trial baseline (in 2012) and endline (in 2014), for cross-sectional surveys, a simple random sample of up to 130 Primary 5 to Primary 7 students per school were invited to consent for a survey interview, and if there were <130 Primary 5 to 7 students then all were invited. Figure 2 describes the timing of the Good School Toolkit delivery and survey assessments. The trial baseline survey data were collected across the 42 schools in 2012 (n=3706, 77% survey response). In 2014, after 18 months of Good School Toolkit implementation in the 21 intervention schools, trial endline data were captured across the 42 schools (n=3820, 93% survey response). Of the students surveyed at the trial endline, 90% (n=3431) agreed to be followed up. These individuals constituted our Wave 1 sample for this study and analysis. The Good School Toolkit intervention was subsequently delivered to 19 of the 21 wait-list control schools from 2015, by which time the Primary 7 students were expected to have left primary school, and 2 wait-list control schools declined to implement the toolkit. Wave 1 adolescents were traced, and Wave 2 survey data collected during 2018 and 2019. A full description of data collection procedures and methods for our Wave 2 survey are reported elsewhere [46].

### Ethics and Consent to Participate

The Contexts of Violence in Adolescence Cohort (CoVAC) study, which encompasses this long-term follow-up, was approved by the London School of Hygiene and Tropical Medicine (6183 and 14768), University of London, Institute of Education, Research Ethics Committee (1091), Uganda Virus Research Institute and Uganda National Council of Science and Technology ethics committees (SS2520 and SS4722). All adult participants and emancipated minors complete voluntary informed written consent procedures prior to involvement in the study. For adolescents under 18 years old who are not emancipated minors, caregivers are provided with information about the study and can verbally opt their children out of participation and, if the caregiver has not opted-out the adolescent, the adolescent provides voluntary informed written assent prior to involvement in the study. Consent procedures have been approved by all ethics committees. During Wave 2, survey referral was offered to adolescents based on predefined criteria agreed with service providers that related to the severity and timing of violence and/or mental health concerns reported. All were offered counseling regardless of what they disclosed. Wave 1 [26] and Wave 2 [46] protocols, including methods and referral procedures [47], are fully documented elsewhere.

### Good School Toolkit Exposure Measures

#### Primary

Participants in 21 trial intervention schools and those in the 21 wait-list control schools are categorized as the original study arms (1) trial and (2) wait-list control. Further subcategories defined within the wait-list control are adolescents who were attending Primary 5 or 6 in the 19 later delivery schools as the (2.1) later delivery exposure group; adolescents who attended one of the schools that did not implement the toolkit or who were attending Primary 7 in one of 19 later delivery schools at Wave 1 (as these students are expected to have left school prior to intervention delivery) as the (2.2) no exposure group (Figure 3).

#### Secondary

Adolescent Good School Toolkit exposure will be further defined using the following data: number of years exposed; time elapsed since exposure; age at exposure; individual survey responses to 2 exposure survey questions on role and participation in the Good School Toolkit intervention; and Raising Voices’ staff assessments of school implementation. Raising Voices’ assessments were captured during in-school visit observations by program staff across 39 schools in 2017 using a standardized scorecard developed by Raising Voices (data were not captured in one trial intervention school that reported they had discontinued the intervention and the 2 wait-list control schools that did not implement the intervention). We will use methods such as exploratory factor analysis to create composite exposure groupings; methods and construction of exposure measures will be fully documented [48,49]. For example, school implementation intensity, length of time exposed, and time since exposure may be used to generate high-, medium- and low-exposure groupings.

### Study Outcome Measures

Study outcome measures are summarized in Table 2.

**Table 2:** Summary of the study outcome measures.

Outcomes			Measure construct	Source
**Violence outcomes:**				
	**Primary outcome:**			
		Peer violence victimization: Self-reported experience of any physical, emotional, and sexual violence between adolescent peers, in the last 12 months.	Question items on physical (6 items), emotional (4 items), and sexual violence (4 items) experienced from a peer, constructed as a binary outcome. Positive response to 1 or more of the 16 items coded=1, negative to all items coded=0. Binary outcomes will also be constructed separately by violence type (physical, emotional, and sexual). A peer is defined as students, coworkers, or community members of a similar age.	Questions adapted from the International Society for the Prevention of Child Abuse and Neglect Child Abuse Screening Tool-Child Institutional [50].
	**Secondary outcomes:**			
		Peer violence perpetration: Self-reported use of physical and emotional violence between adolescent peers, in the last 12 months.	Questions items on physical (2 items) and emotional violence (3 items) use against a peer, constructed as a binary outcome. Positive response to 1 or more of the 5 items coded=1, negative to all items coded=0. Binary outcomes also constructed separately by violence type (physical and emotional). A peer is defined as students, coworkers, or community members of a similar age.	Questions adapted from the International Society for the Prevention of Child Abuse and Neglect Child Abuse Screening Tool-Child Institutional [50]
		Intimate-partner violence victimization: Self-reported experience of physical, emotional, and sexual intimate-partner violence among ever partnered adolescent women, in the last 12 months.	Question items on physical (3 items), emotional (8 items), and sexual violence (4 items) experienced from a partner, constructed as a binary outcome. Positive response to one or more of the 15 items coded=1, negative to all items coded=0. Binary outcomes also constructed separately by violence type (physical, emotional, and sexual). Intimate partners include boy/girlfriends, husbands/wives, and casual dating partners.	Adapted from the World Health Organization multicountry study on women’s health and domestic violence against women [51], and the Conflict in Adolescent Dating Relationships Inventory [52,53]
		Intimate-partner violence perpetration: Self-reported use of physical and emotional intimate-partner violence, among ever partnered adolescent men, in the last 12 months.	Question items on physical (2 items) and emotional (3 items) violence use constructed as a binary outcome. Positive response to one or more of the 5 items coded=1, negative to all items coded=0. Binary outcomes also constructed separately by violence type (physical and emotional). Intimate partners include boy/girlfriends, husbands/wives, and casual dating partners.	Adapted from the World Health Organization multicountry study on women’s health and domestic violence against women [51], and the Conflict in Adolescent Dating Relationships Inventory [52,53]
**Attitudes outcomes:**				
		Student acceptance of teacher violence	3 items will be constructed as a continuous outcome. Each response option will be assigned a score of 0–3. Scores summed and modeled as continuous score range 0 (low) to 9 (high acceptability)	Measure from the Good Schools Study [41]
		Adolescents attitudes toward gender relations	18 items will be initially explored as one construct to generate a continuous outcome. Response options numerally coded 0-3 and individual scores calculated, ranging from 0-54. Attitudes toward domestic violence from Gender Equitable Men subscale 5 items will be explored as a continuous outcome and as single question items.	Gender Equitable Men scale, Uganda version [54]
**Outcomes supportive of resilience:**				
		Agency	13 items, 3 independent dimensions, Internality (4 items), Powerful Others (4 items), and Chance (5 items). Each subscale will be initially explored as separate continuous outcome measures, response options will be assigned a score 0-3, summed and modeled as continuous scores.	Adapted Internality, Powerful Others, and Chance measure of locus of control, short-form
		Self-regulation	13 items will be initially explored as one construct (one continuous outcome), and further explored as a 2-dimensional measure, with restraint and impulsivity modeled as separate continuous outcomes [55,56].	Brief Self-Control Scale [57]
		Social assets	Continuous outcome, 8 items with response options: 0 not true, 1 somewhat true, and 2 certainly true. scores summed and modeled as continuous score range 0-16	Constructed from subsets of the Strengths and Difficulties Questionnaire [58,59]
		Psychological assets	Continuous outcome, 7 items, reverse coded: 2 not true, 1 somewhat true and 0 certainly true. Response options assigned a score 0-2, range: 0-14	Constructed from subsets of the Strengths and Difficulties Questionnaire [58,59]
		Peer connectedness	2 items, continuous outcome generated, response options numerally coded 0-3 and individual scores calculated, range 0-6.	Questions adapted from scales used in adolescent health behavior surveys [60] and used in Good Schools Study. Response options: all the time, most of the time, sometimes, or never
**Retention in school outcomes**				
		Staying in school	Last school grade completed will be assessed in relation to current age and grade at Wave 1, for example, binary outcomes: completion of grade Primary 7 (yes=1, no=0), transition to secondary school measured as completion of grade Secondary 1 (yes=1, no=0)	Not applicable

The primary outcome is peer violence victimization. A peer is defined as students, coworkers, or community members of a similar age. Adolescents’ self-reported experience of peer violence in the last year will be measured using an adapted subset of questions from the International Society for the Prevention of Child Abuse and Neglect Child Abuse Screening Tool—Child: Institutional [61]. Questions were previously piloted and used in the Wave 1 survey [32]. For this analysis, peer violence is defined as violence from students, coworkers, or community members of a similar age. Experience of past-year peer violence will be constructed as a composite binary measure, with positive responses to one or more question items on acts of physical, emotional, and sexual violence coded as 1, and no acts coded as 0.

Secondary violence outcomes will be constructed as single and composite binary measures—as described above—for the primary outcome. Secondary violence outcome measures include past-year self-reported use of peer violence, girls’ experience and boys’ use of intimate-partner violence, measured using question items from the WHO multicountry Study on Women’s Health and Domestic Violence [51] and Conflict in Adolescent Dating Relationships Inventory [52,53,62] adapted and used during Wave 1. Intimate partners include boy/girlfriends, husbands/wives, and casual dating partners. Self-reported intimate-partner violence will be explored as a combined measure and separately by violence type (emotional, physical, and sexual).

We will measure attitudes toward teacher violence with a 3-item measure developed for the Good Schools Study and used during the Wave 1 survey [41]. A shortened 18-item Gender Equitable Men scale, previously validated among Ugandan adolescents [54], will be used to measure gender-equitable attitudes. Question items and subsets of questions, including attitudes toward domestic violence, will be explored as single items and composite outcomes. Response options for all attitude questions are on a 4-point Likert-type scale.

Factors supportive of resilience will be measured, including peer connectedness [41], social assets, and psychological assets [63], these 3 measures had adequate internal consistency at Wave 1 [58,59,64]. The response options were on a 3-point scale for the asset questions and a 4-point Likert-type scale for the connectedness questions. Furthermore, we will explore using the Internality, Powerful Others, and Chance measure of locus of control, as a proxy measure of agency [65,66], and the Brief Self Control Scale [57,67], as a proxy to measure self-regulation. As neither Internality, Powerful Others, and Chance nor Brief Self Control Scale measures have been validated among Ugandan adolescents, questions were forward and backward translated by bilingual researchers, concepts were reviewed by a user group of Ugandan adolescents, wording was iteratively adapted through cognitive interviews with 8 to 15 adolescents, and the questions were pilot tested before inclusion in the Wave 2 survey questionnaire. If the outcomes measures for either have low internal consistency (Cronbach α<.65) then exploratory factor analysis will be considered to generate factors scores and groupings.

To measure retention in school, we will capture last school grade completed and assess this in relation to expected grade based on school grade at Wave 1 and current age.

### Potential Confounders and Effect Modifiers

The following factors have been identified a priori as potential confounders or effect-modifiers to the exposure-outcome relationships of interest: biological sex and the number of meals eaten the previous day (as a proxy for socioeconomic status that is expected to be associated with staying in the study school throughout intervention delivery and associated with later violence outcomes). In addition, and as appropriate for specific exposure-outcome relationships, the following variables have been identified as potential effect-modifiers or confounders measured at Wave 1: school grade, family connectedness, and experiences of violence outside of school [68,69].

### Power to Detect a Difference

We anticipate that our trial exposure group will be 50% (n=1201), and no exposure group 19% (n=456), of the total Wave 2 sample (assuming a conservative 70% follow-up, 2402/3431). We estimate 50% [2] of our no exposure group will report past-year experiences of physical, emotional, or sexual violence from a peer at Wave 2. In this case, the smallest difference we can detect between no exposure and trial exposure groups, assuming 70% follow-up, an α of .5 and power of .8, would be an 8% difference between groups (OR 0.73).

### Data Management

All Wave 1 and Wave 2 survey data were captured, transmitted, and stored on a secure server using Open Data Kit. Raising Voices school-level implementation assessment data were initially captured on standardized Excel (Microsoft Inc) spreadsheets scorecard. All data will be transferred to Stata 15 (StataCorp LLP) for all data management processes and analysis. A data set containing Wave 1 data (2014) will be individually linked with Wave 2 data (2018-2019) and merged with school-level aggregate data from the Good Schools Study baseline survey (2012) and Raising Voices school-level program implementation assessment scorecard data (2017), to create one data set for analysis.

### Analysis Plan

Adolescents’ sociodemographic characteristics will be described for Wave 1 and Wave 2 and by intervention-exposure groupings. Descriptive statistics for continuous variables will include the number of observations, mean, and standard deviation (or median and interquartile range as appropriate). Categorical variables will be presented as numbers and percentages. We will formally test for differences in characteristics between those who participated in Wave 1 and 2 compared to Wave 1 only (eg, age, sex, number of meals eaten the day before, school grade, and original study arm). We will account for clustering by original primary school (by using the Stata *Svy* command) and Taylor linearized variance estimation to calculate standard errors, present corrected person chi-square for categorical data, and associated *P* values and 95% confidence intervals. Together, these will be considered as evidence toward differential attrition [70].

### Regression Analysis Strategy

All analysis is exploratory and hypotheses generating rather than testing. To explore if the intervention influences later outcomes, we will compare across the trial and no exposure groups (1 vs 2.2) and original study arms (1 vs 2) first including all 42 schools and then repeated, dropping the 2 nonimplementation schools. To further explore differences between levels of exposure, we plan to compare between trial and the later delivery groups (1 vs 2.1) and across secondary exposure groupings (Figure 3). We will account for clustering by including original study primary school as a random effect in mixed-effect regression models [71]. Binary outcomes will be analyzed using mixed-effect logistic regression, with effect size presented as odds ratios. Continuous outcomes will be analyzed using mixed-effects linear regression, with effect size presented as mean difference. All unadjusted and adjusted analyses will include baseline school-level mean of outcomes, where available [72]. Adjusted models will additionally include covariates identified as potential confounders. All measures of effect will be presented with 95% confidence intervals and *P* values. Size and direction of effect will be considered along with *P* values when assessing the strength of associations [70]. Appropriate methods will be used to analyze numeric outcomes that are not normally distributed. For example, bias-corrected 95% confidence intervals will be estimated using nonparametric bootstrapping. The presence of effect modification will be assessed by comparing models fitted with and without an interaction term. Likelihood ratio tests with *P* values<.1, along with the direction and size of effect for each group, will be considered as evidence for or against modification. If there is evidence of modification, then stratum-specific effects will be calculated [73].

For self-reported exposure, propensity score–matching will be used to account for the likelihood that self-reported exposure is confounded by an individual’s propensity to report exposure. We will estimate what factors predict toolkit exposure using regression analysis [29], then calculate predicted probability of exposure and group adolescents with similar propensity scores. The analysis will compare outcomes across these propensity score exposure groups, including the score as a covariate in regression models [72].

For the primary peer-violence victimization outcome, we will present unadjusted and adjusted mixed-effect logistic regression models exploring the association between intervention exposure measures and peer-violence victimization.

For the secondary outcomes, we will present unadjusted and adjusted mixed-effect logistic or linear regression models exploring the association between intervention exposure measures and secondary outcomes. The effect of intervention exposure on secondary outcome measures will be assessed by the size of effects and accompanying *P* value and 95% confidence intervals, as well as expected direction and consistency across results.

## Results

Data collection started in 2014 (Wave 1) and data collection was completed in 2019 (Wave 2). Data analysis has not been conducted.

## Discussion

### Principal Findings

This is the first study that evaluates the long-term impacts of an effective primary school violence-prevention intervention in a resource-constrained setting, where the majority of adolescents transition out of school after primary education. Guided by our conceptual framework, we plan to explore how long-term effects on violence and other outcomes may vary across schools and adolescents to build a picture of what works and for whom. This analysis will shed light on the generalizability of effects to inform intervention strengthening, future targeting, and generate hypotheses on potential transferability to other contexts. For instance, if there are no long-term impacts on retention in school, violence outcomes, or factors supportive of resilience, then this will suggest that further efforts are required to reinforce and sustain positive effects. Findings might point toward the need for complementary interventions in secondary schools or targeting family support and the home environment. Little is known about whether capabilities gained in primary school can be maintained through transition into new schools or the workplace, nor whether experiencing improved relationships and school connectedness in a safer primary school environment can positively influence future peer and intimate relationships among older Ugandan adolescents. Our findings will highlight important areas for further qualitative and quantitative inquiry to better understand how school interventions shape violence and other outcomes through adolescent transitions.

### Limitations

This protocol focuses on adolescent long-term outcomes, therefore, whether the intervention is maintained in primary schools and the influence of secondary school environments on outcomes are questions beyond the scope of the analysis. Our quasi-experimental nonrandomized design utilizes the trial delivery and later delivery of the intervention to schools and makes use of an unexposed comparison group that arose from the process and timing of delivery. As in other nonrandomized studies, confounding is of concern, as comparison groups differ regarding factors other than exposure to the intervention. To address this, we have a documented conceptual framework, plan to control for potential observed confounding in our analyses, and will consider the consistency of effects when interpreting results [74]. We expect to gain further insight into the toolkit’s long-term effectiveness by comparing effects across original study arms in addition to across other exposure groupings.

This is an unblinded study, where both participants and researchers—including those leading the analysis—are aware of the intervention delivery. Selection bias may be introduced at Wave 1 as 10% of students in the original trial endline did not agree to follow-up. Differences in retention at Wave 2 could be associated with our outcomes of interest or with toolkit exposure, we will test for differences and, where possible, apply appropriate statistical methods to take account for such differences.

### Conclusion

This analysis will be the first to examine the long-term effects of a whole-school violence-prevention intervention in a resource-constrained setting. If results are positive, this indicates that primary school interventions are promising platforms to institute lasting widespread change. This is especially relevant to alleviate the burden of many adverse outcomes of early exposure to violence in Uganda and other countries.

## Abbreviations

CoVAC: Contexts of Violence in Adolescence Cohort study
OR: odds ratio
WHO: World Health Organization
